# Neuroelectrical Correlates of Trustworthiness and Dominance Judgments Related to the Observation of Political Candidates

**DOI:** 10.1155/2014/434296

**Published:** 2014-08-24

**Authors:** Giovanni Vecchiato, Jlenia Toppi, Anton Giulio Maglione, Elzbieta Olejarczyk, Laura Astolfi, Donatella Mattia, Alfredo Colosimo, Fabio Babiloni

**Affiliations:** ^1^Department of Physiology and Pharmacology, “Sapienza” University, Piazzale Aldo Moro 5, 00185 Rome, Italy; ^2^Department of Computer, Control, and Management Engineering, “Sapienza” University, Viale Ariosto 5, 00185 Rome, Italy; ^3^IRCCS Fondazione Santa Lucia, Via Ardeatina 306, 00179 Rome, Italy; ^4^Department of Anatomy, Histology, Forensic Medicine and Orthopedics, “Sapienza” University, Via Borelli 50, 00185 Rome, Italy; ^5^Nałęcz Institute of Biocybernetics and Biomedical Engineering, Polish Academy of Sciences, 4 Trojdena Street, 02-109 Warsaw, Poland

## Abstract

The present research investigates the neurophysiological activity elicited by fast observations of faces of real candidates during simulated political elections. We used simultaneous recording of electroencephalographic (EEG) signals as well as galvanic skin response (GSR) and heart rate (HR) as measurements of central and autonomic nervous systems. Twenty healthy subjects were asked to give judgments on dominance, trustworthiness, and a preference of vote related to the politicians' faces. We used high-resolution EEG techniques to map statistical differences of power spectral density (PSD) cortical activity onto a realistic head model as well as partial directed coherence (PDC) and graph theory metrics to estimate the functional connectivity networks and investigate the role of cortical regions of interest (ROIs). Behavioral results revealed that judgment of dominance trait is the most predictive of the outcome of the simulated elections. Statistical comparisons related to PSD and PDC values highlighted an asymmetry in the activation of frontal cortical areas associated with the valence of the judged trait as well as to the probability to cast the vote. Overall, our results highlight the existence of cortical EEG features which are correlated with the prediction of vote and with the judgment of trustworthy and dominant faces.

## 1. Introduction

A growing number of research laboratories are involved in investigating cerebral areas activated during the observation of pictures showing politicians, as well as videos supporting them during electoral campaigns. In the pioneering study in the field, faces of coupled candidates for political elections in the USA Senate were presented for less than a second [1]. Judgments based on such a “superficial” observation could predict the election results, being linearly correlated with the candidate's margin of victory. In other words, emotional inferences formed on the basis of the observation of a face for less than a second could even overcome rational considerations about the hypothetical future work of the candidate. The consequence of this result is that the appeal of a politician's face might be the principal factor for the citizen's choice, even more than rational considerations. In fact, such a phenomenon has also been confirmed by subsequent studies [2], suggesting that the scenic presence by itself mostly influence the decision of vote. These results, concerning the behavioural psychology, are surprising if we think about the USA midterm elections of 2006 when candidates and their supporting groups spent about 1 billion dollars in advertisements in order to inform electors about their political affiliation, qualities, and ideas to promote [3]. Afterwards, researchers moved to understand whether this first impression is more due to positive effects (i.e., pleasantness, adaptation to particular a priori requirements) or negative ones (i.e., negative judgement about pleasantness implicitly extended to the competence field) [4]. The surprising result of this study was that an emotionally “negative” judgement towards a candidate (as being “less appealing” than the opponent) is a prevailing reason for his defeat, even in a contest of simulated elections. In addition, the cerebral activity generated from an emotional state of “rejection” of the candidate is completely different from the one generated from an emotional state of acceptation or satisfaction [5]. Neuropolitical studies have found that the activation of emotion-related areas in the brain is linked to political preferences, identifying the neural correlates in the prefrontal cortices of changes in political attitudes toward others that are linked to social cognition [6]. In particular, in a study performed by functional magnetic resonance imaging (fMRI), Spezio and colleagues [5] revealed that activation in the insula/parainsula and anterior cingulate cortex (ACC) correlates with election loss in a simulated voting paradigm.

Another study [7] investigated how political party affiliation and political attitudes modulate neural activity while viewing faces of presidential candidates. They found that viewing the candidate from the opposing political party produced signal changes in cognitive control circuitry in the dorsolateral prefrontal cortex and anterior cingulate, as well as in emotional regions such as the insula and anterior temporal poles.

From the traditional political research that is performed without any neurophysiological measurements, it was already known that the negative vote plays an important role in the final vote decision [8–10]. In this context, it is important to understand the role of people's emotional behaviour after observing a politician compared with the one of a whole electoral campaign.

It is very well known that the hemodynamic measurements of the brain activity have the spatial resolution of the order of cubic mm, being capable of detecting activations also in deep brain structures such as amygdala and nucleus accumbens. However, the lack of time resolution, due to the delay of the cerebral blood flow increment after the exposition to the stimuli, makes the fMRI unsuitable to follow the brain dynamics. For this reason, other authors also adopt different tools such as magnetoencephalography (MEG). This technique is sensitive to changes of magnetic fields that are induced by the electrical brain activity, and it is able to detect rapid changes of the neural activity on a temporal scale of milliseconds and on a spatial scale of centimetres [11].

It is worth noting that in the past several studies electroencephalography (EEG) was also used as brain imaging tool for the analysis of brain activity during the observation of faces and emotional stimuli (for a review see [12]). High-resolution EEG technology has been developed to enhance the poor spatial information content of the EEG activity in order to detect the brain activity with a spatial resolution of a squared centimetre and the unsurpassed time resolution of milliseconds [13–17].

Recently, it was realized that the functional connectivity networks [18, 19] estimated from brain-imaging data obtained by EEG, MEG (magnetoencephalography), and fMRI can be investigated using graph theory [20–29]. Since a graph is a mathematical representation of a network that has been essentially reduced to nodes and connections between them, the use of a graph-theory approach is potentially relevant and useful to quantify and describe the degree and modality of communication among different cerebral areas, as first demonstrated on a set of anatomical brain networks [30, 31].

The use of EEG allows following the brain activity on a millisecond base, but it has the problem that the recorded EEG signals are mainly due to the activity generated in the cortical structures of the brain. In fact, the electromagnetic activity elicited by deep structures advocated for the generation of emotional processing in humans is almost impossible to gather from usual superficial EEG electrodes [13, 32]. It has been underlined that a positive or negative emotional processing of the commercial advertisements is an important factor for the formation of stable memory traces [6]. Hence, it became relevant to infer the emotional engage of the subject by using indirect signs for it. In fact, indirect signs of emotional processing could be gathered by picking variations of the activity of the anatomical structures linked to the emotional processing activity in humans, such as the activity of sweat glands on the hands and/or the variation of the heart rate [33]. In particular, by monitoring autonomic activity using devices able to record the variation of the skin conductivity (galvanic skin responses (GSR)) and the heart rate (HR), it is possible to assess the “internal” emotional state of the subject [34]. In fact, galvanic skin response (GSR) activity is actually viewed as a sensitive and convenient measure of sympathetic arousal associated with emotion, cognition, and attention [35]. Studies using functional imaging techniques [35, 36] have related the generation and level of electrodermal activity to specific brain areas. These specific regions are the ventromedial prefrontal cortex, orbitofrontal cortex, left primary motor cortex, and the anterior and posterior cingulate, which have been shown to be associated with emotional and motivational behaviours [35, 36]. Such findings indicate the close association of peripheral and central measures of arousal, emphasising the close connections between electrodermal activity, arousal, attention, cognition, and emotion. In addition, the link between the heart rate (HR) or the heart rate variability (HRV) and the sympathovagal balance has also been suggested [37–39].

In this study, we were interested in analyzing the neuroelectrical and autonomic activity elicited during the observation of real politicians' faces who actually participated in municipal and regional elections held in Italy in the 2004 and 2008. The aim of the present research was to investigate the EEG activity of a group of twenty healthy subjects while they were asked to both vote in simulated elections and give a judgment about personal traits of real politicians shown. By means of high-resolution EEG technique, we mapped statistical differences of cortical spectral activity elicited during the observation of candidates while subjects were asked to give a judgment on dominance, trustworthiness traits, and a preference of vote on the faces seen. To measure both the electrical brain activity and the autonomic responses, we used simultaneous EEG, GSR, and HR measurements during the whole experiment. Moreover, analysis of the electrodermal activity and the heart rate variability related to such as stimuli has also been performed, along with the study of the participant's choice. Thus, these measurements have been able to analyze the proposed social paradigm from several perspectives, by collecting the explicit judgment of simulated voters and by analyzing their cognitive and emotional aptitude through the estimation of cortical spectral activity and the related functional connectivity as well as parameters of the autonomic nervous system.

In particular, the objectives of the present study can be summarized through the formulation of the following experimental questions.

1. Is it possible to predict the elections outcome through the subjects' rapid and explicit judgment of dominance and trustworthiness traits?
2. Are there any EEG features able to predict subjects' preference of vote? Are there any correlations between the cortical functional activity and the judgment of trustworthiness and dominance?
3. Is there any autonomic signature correlating with dominance and trustworthiness traits of politicians' faces?

## 2. Material and Methods

### 2.1. Experimental Design and Stimuli

Twenty healthy volunteers (mean age 26.7 ± 3.7 years; 9 women) have been recruited for this experiment. Participants were asked to vote for real political candidates who run against each other during the municipal and provincial elections of the years 2004 and 2008. Pictures of the winner and the runner-up were collected from various Internet sources (e.g., website of the Italian Ministry of the Interior and local media sources). Each photo has been processed by a professional photographer in order to convert it into black and white, balance contrast, and luminance. Some races were unusable because of the low resolution of candidate's photos. For the remaining 70 races, the image of each politician was cropped to a common size (250 × 361 pixels) and placed on a grey background. All faces have been adapted to fit the same size and to occupy the visual field of around 5.9 × 9.1 degrees on a screen with dimension of 337 × 270 mm and resolution of 1024 × 768 pixels. Participants were sitting at 80 cm from the screen.

**Figure 1:**
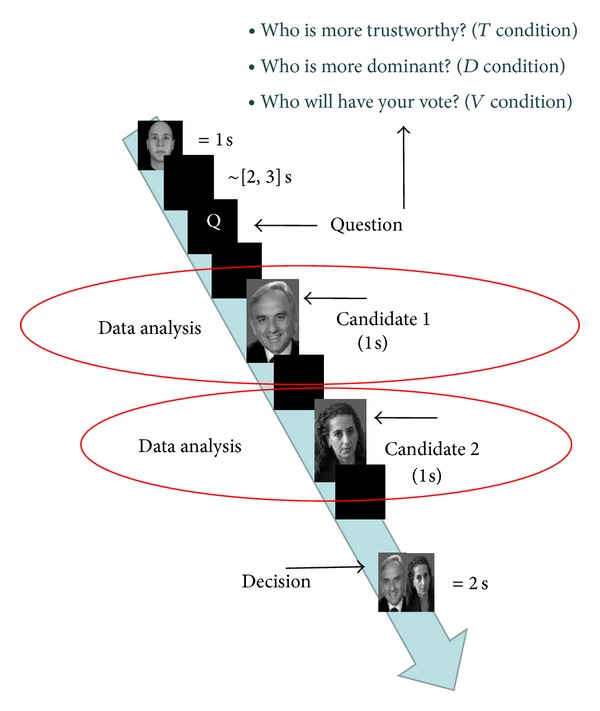
Epochs of a typical trial. Each trial began with the image of a neutral face (1 s) to allow a washout of the neurophysiological variables. Afterwards, subjects were asked to express a judgment about the subsequent political candidates related to traits of trustworthiness, dominance, and preference of vote which were randomized among trials (3 s). Then, each single face of the political candidates racing for the same election appeared separately and in random order (1 s) among subjects and judgment. Finally, both faces were presented together for the decision stage (2 s). All stimuli were intermingled by a black screen (~[2, 3] s).

Our subjects were asked to give a preference of vote (vote condition) according to the images of political candidates. Moreover, subjects were also asked to judge the same politicians for their traits of dominance and trustworthiness (dominance and trustworthiness conditions, resp.). At the beginning of each trial, a neutral face was shown [40]. Subsequently, the question that subjects were asked was shown, followed by the pictures of the two candidates presented one by one for 1 s, intermingled by blank screens of length 2-3 s (random variable uniformly distributed). Finally, the pictures of both politicians, side by side, were presented for a maximum of 2 s. During that time, subjects were asked to give their vote and judgment according to the former question, by pressing the left/right arrow button on the keyboard. If they did not express any preference within 2 s interval, the trial will be classified as an abstention. The order of presentation of the candidates, as well as their position of appearance in the trial, was fully randomized to show each pair of faces in all three conditions. Epochs of a representative trial are illustrated in Figure 1.

### 2.2. Behavioural Data

For each couple of politicians and judgment (vote, dominance, and trustworthiness), we collected the subject's choice. For each experimental condition, we calculated the percentage of correct prediction: each judgement has been compared with the outcome of the election through the following formula:

(1) $$\begin{array}{c} \text{PR}=\frac{\sum_{i=1}^{N}\delta \left(V_{i},\tilde{V}\right)}{N}\times 100, \end{array}$$

where *δ* is the Kronecker delta, *V*_*i*_ is the given vote or judgment explicitly expressed by subjects, and $\tilde{V}$ is the outcome of the real elections. If the subject chose the politician who actually won the race, we considered that the related outcome of the simulated race has been correctly predicted. Subjects were not aware of the outcome of the real elections. At the end of the experiment, participants were asked to report if they recognized some of the seen faces and if they knew the real outcome of the elections. In such a case, the related trials were discarded from the analysis. Repeated-measures analysis of variance (ANOVA) was performed to assess differences between percentage of correct prediction among judgements. For each couple of judgment (vote/dominance, vote/trustworthiness, and dominance/trustworthiness) the Pearson's correlation has been calculated between the rates of correct prediction of election races.

### 2.3. EEG Recordings and Preprocessing

The cerebral activity has been recorded with a 61-channel system (Brain Amp, Brainproducts GmbH, Germany) with a sampling rate of 200 Hz. The EEG signals have been band pass filtered at 1–45 Hz and depurated of ocular artefacts by employing the independent component analysis (ICA): the components due to eye blinks and ocular movements have been detected by eye inspection and then removed from the original signal. The collected data have been segmented in order to analyze the neurophysiological activity elicited during the 1 s observation of the politicians. Thus, each segment was 1 s long for the EEG signal. All of them have been classified into six subsets. They were associated, for instance, with the observation of faces which have casted the vote (*V* ^+^) and those which did not (*V* ^−^). In the same way, we grouped the segments related to faces which have been judged more/less dominant (*D* ^+^/*D* ^−^) and more/less trustworthy (*T* ^+^/*T* ^−^), respectively. For each dataset, a semiautomatic procedure has been also adopted to reject trials presenting muscular and other kinds of artefacts. Only artefacts-free trials have been considered for the following analysis. The extra-cerebrally referred EEG signals have been transformed by means of the common average reference (CAR). The individual alpha frequency (IAF) has been calculated for each subject in order to define six bands of interest according to the method suggested in the literature [41]. Such bands were in the following reported as IAF+*x*, where IAF is the individual alpha frequency, in Hertz, and *x* is an integer displacement in the frequency domain which is employed to define the band. In particular, we defined the following six frequency bands: theta (IAF−6, IAF−2), for example, theta ranges between IAF−6 and IAF−2 Hz, alpha (IAF−2, IAF+2), beta (IAF+2, IAF+15), and gamma (IAF+15, IAF+30).

### 2.4. Estimation of Cortical Power Spectral Density

The high-resolution EEG technologies [13, 15, 17, 42–44] have been adopted in order to obtain an estimation of the cortical power spectral density (PSD). The scalp, skull, and dura mater compartments were built by using 1200 triangles for each structure, and the boundary element model was then employed to solve the forward electromagnetic problem. For each subject, the electrodes disposition, in terms of coordinates, on the scalp surface was calculated through a nonlinear minimization procedure [45]. The cortical model consisted of 7953 dipoles uniformly distributed on the cortical surface, and the estimation of the current density strength for each dipole was obtained by solving the electromagnetic linear inverse problem according to the minimum norm solution as described in the previous papers [17, 45–49]. Each dipole was modeled to be perpendicular to the cortical surface. The cortical power spectral density (PSD), calculated with the Welch method [50], has been computed for each equivalent cortical current dipole of the realistic average head model (Colin template, 7953 cortical dipoles) by solving the electromagnetic linear inverse problem. This procedure has allowed us to obtain a measure of PSDs values for each cortical dipole and for each trial for all datasets. In order to take into account subjects' personal baseline activity, we used the neurophysiological signals (mean and standard deviation) related to the observation of the neutral face to transform into *z*-score variables the values of spectral power of vote, dominance, and trustworthiness datasets according to the following formula:

(2) $$\begin{array}{c} Z_{V,D,T}=\frac{X_{V,D,T}-\mu_{N}}{\sigma_{N}}, \end{array}$$

where *Z*_*V*,*D*,*T*_ is the *z*-score value related to the vote, dominance, and trustworthiness dataset, whereas *μ*_*N*_ and *σ*_*N*_ are mean and standard deviation related to the neutral face dataset.

To improve the normality properties of such distributions, all variables have been transformed into normal distributions [51]. Finally, for each of the three conditions, we used the *t*-test (*P* < 0.05) to compare the transformed PSD distribution, for each equivalent cortical current dipole and the aforementioned frequency bands of interest, and adopted the false discovery rate correction for multiple comparisons [49].

### 2.5. Partial Directed Coherence

The partial directed coherence PDC [52] is a full multivariate spectral measure used to determine the directed influences between any given pair of signals in a multivariate data set. PDC is a frequency domain representation of the existing multivariate relationships between simultaneously analyzed time series that allows the inference of functional relationships between them. This estimator was demonstrated to be a frequency version of the concept of Granger causality [53], according to which a time series *x*[*n*] can be said to have an influence on another time series *y*[*n*] if the knowledge of past samples of *x* significantly reduces the prediction error for the present sample of *y*. In this study, the PDC technique was applied to the set of several regions of interest (ROIs) in which the cortical surface has been segmented according to Brodmann areas (BAs). Namely, we defined the following bilateral areas of interest: 10, 8, 5, 7, 37, 19, 9/46, 21/22, and 41/42. This choice of the selected ROIs can be justified at the light of the results provided by the published literature [5–7] showing how these cerebral areas are involved during the observation of political candidates (photos and videos) as well as in their judgment. Due to computational limitations of the PDC method, we could not cover the whole cortical surface but only select the most representative regions. However, this cortical segmentation has not been taken into account for the PSD analysis because we preferred to exploit the enhance of the spatial resolution and to highlight changes of activity of single equivalent cortical current dipoles estimated. In such a way, a total of eighteen ROIs has been taken into account for the subsequent functional connectivity analysis performed via partial directed coherence (PDC) and graph-theory metrics. In the following, we denote the ROIs signals *S*:

(3) $$\begin{array}{c} S={\left[s_{1}\left(t\right),s_{2}\left(t\right),\ldots ,s_{N}\left(t\right)\right]}^{T}. \end{array}$$

Let us to suppose that the following MVAR process is an adequate description of the data set *S*:

(4) $$\begin{array}{c} \overset{p}{\underset{k=0}{\sum }}\Lambda_{k}S\left(t-k\right)=E\left(t\right)\;\text{with}\;\;\Lambda_{0}=I. \end{array}$$

In this expression, *E*(*t*) = [*e*_1_(*t*), *e*_2_(*t*),…, *e*_*N*_(*t*)]^*T*^ is a vector of multivariate zero-mean uncorrelated white noise process, Λ_1_, Λ_2_,…, Λ_*p*_ are the *N* × *N* matrices of model coefficients, and *p* is the model order, chosen, in this case, by means of the Akaike information criteria (AIC) for MVAR processes [54]. Once an MVAR model is adequately estimated, it becomes the basis for subsequent spectral analysis. In order to investigate the spectral properties of the examined process, (2) is transformed to the frequency domain:

(5) $$\begin{array}{c} \Lambda \left(f\right)S\left(f\right)=E\left(f\right), \end{array}$$

where

(6) $$\begin{array}{c} \Lambda \left(f\right)=\overset{p}{\underset{k=0}{\sum }}\Lambda_{k}e^{-j2\pi f\Delta tk}, \end{array}$$

and Δ*t* is the temporal interval between two samples.

It is then possible to define PDC as

(7) $$\begin{array}{c} \pi_{ij}\left(f\right)=\frac{\Lambda_{ij}\left(f\right)}{\sqrt{\sum_{k=1}^{N}\Lambda_{kj}\left(f\right)\Lambda_{kj}^{*}\left(f\right)}}. \end{array}$$

Such formulation was derived by the well-known concept of partial coherence [52]. The PDC from *j* to *i*, *π*_*ij*_(*f*), describes the directional flow of information from the signal *s*_*j*_(*n*) to *s*_*i*_(*n*), whereupon common effects produced by other electrodes *s*_*k*_(*n*) on the latter are subtracted leaving only a description that is exclusive from *s*_*j*_(*n*) to *s*_*i*_(*n*).

PDC values are in the interval [0,1] and the normalization condition

(8) $$\begin{array}{c} \overset{N}{\underset{n=1}{\sum }}{\left|\pi_{nj}(f)\right|}^{2} \end{array}$$

is verified. According to this condition, *π*_*ij*_(*f*) represents the fraction of the time evolution of electrode *j* directed to electrode *i*, as compared to all of *j*'s interactions to other electrodes.

Even if this formulation derived directly from information theory, the original definition was modified in order to give a better physiological interpretation to the estimation results achieved on electrophysiological data. In particular, two modifications have been proposed. First, a new type of normalization, already used for another connectivity estimator such as directed transfer function [55], was introduced by dividing each estimated value of PDC for the root squared sums of all elements of the relative row, and then a squared version of the PDC was introduced [56]:

(9) $$\begin{array}{c} {\text{sPDC}}_{ij}\left(f\right)=\frac{{\left|\Lambda_{ij}\left(f\right)\right|}^{2}}{\sum_{m=1}^{N}{\left|\Lambda_{im}\left(f\right)\right|}^{2}}. \end{array}$$

The better performances of sPDC have been demonstrated in simulation studies which revealed reduced error levels both in the estimation of connectivity patterns on data characterized by different lengths and SNR and in distinction between direct and indirect paths [56]. Such formulation was used in this study for the estimation of functional connectivity.

### 2.6. Statistical Validation of Connectivity Patterns

Random fluctuations of signals, induced by environmental noise, could lead to the presence of spurious links in the connectivity estimation process. In order to avoid such false connections, it is necessary to apply a method for the statistical validation of estimated connectivity patterns. In order to assess the significance of the estimated connectivity patterns, the value of effective connectivity for a given pair of electrodes, obtained by computing PDC [57, 58], must be statistically compared with a threshold level which is related to the lack of transmission between considered ROIs (null hypothesis). Threshold values were estimated using asymptotic statistic [59], a recently introduced method based on the assumption that PDC in the null case follows a *χ* ^2^ distribution [60]. The statistical threshold for the null case is achieved by applying a percentile, related to a given significance level, on a *χ* ^2^ distribution derived by means of Monte Carlo method directly from the data. The high accuracy of the asymptotic statistic method in the assessment process has been demonstrated in a simulation study in which this new method was compared with the shuffling procedure [61, 62], a time consuming methodology currently available in the functional connectivity field [63].

The statistical validation process had to be applied on each couple of signals for each frequency sample. This necessity led to the execution of a high number of simultaneously univariate statistical tests with evident consequences in the occurrence of type I errors. The statistic theory provides several techniques that could be usefully applied in the context of the assessment of connectivity patterns in order to avoid the occurrence of false positives [64]. In particular, we chose the false discovery rate (FDR) method [65, 66] because it has been demonstrated to be a good compromise in preventing both type I and type II errors occurred during connectivity estimation [67, 68].

After the validation process, the PDC estimation is averaged within four frequency bands defined according to individual alpha frequency (IAF) [41] in order to take into account the variability among subjects of the alpha peak in the spectrum. The range for each frequency band is theta [IAF−6; IAF−2], alpha [IAF−2; IAF+2], beta [IAF+2; IAF+15], and gamma [IAF+15; IAF+30]. PDC connections of the three judgments (*V*, *T*, and *D*) have been separately compared between valence (+, −), for each band of interest, by performing multiple Student's *t*-test on ROIs, FDR corrected.

### 2.7. Graph Theory

A graph consists of a set of vertices (or nodes) and a set of edges (or connections) indicating the presence of some sort of interaction between the vertices. The adjacency matrix *A* contains the information about the connectivity structure of the graph. When a directed edge exists from the node *i* to *j*, the corresponding entry of the adjacency matrix is *A*_*ij*_ = 1; otherwise, *A*_*ij*_ = 0. The existence of an edge is stated on the basis of its statistical significance assessed by means of asymptotic statistic approach. The higher reliability of the statistical approach for extracting the adjacency matrix has been demonstrated in [67] where a detailed comparison with the empirical methods is provided. In graph theory, a path or a walk is a sequence of vertices in which from each of its vertices, there is an edge to the next vertex in the sequence. Such adjacency matrix can be used for the extraction of salient information about the characteristic of the investigated network by defining several indexes based on the elements of such matrix.

*Density.* Density is the fraction of present connections to possible connections. Connection weights are ignored in calculations. Density is defined as follows:

(10) $$\begin{array}{c} D=\frac{\sum_{i=1}^{N}\sum_{j=1}^{N}A_{ij}}{N\left(N-1\right)}\times 100, \end{array}$$

where *A*_*ij*_ represents the entry *ij* of the adjacency matrix *A*.

For each judgment (*V*, *T*, and *D*), we performed a repeated measure ANOVA with factor BAND (theta, alpha, beta, and gamma) to compare the density value among bands of interest.

*Degree.* The degree of a node is the number of links connected directly to it. In directed networks, the indegree is the number of inward links and the outdegree is the number of outward links. Connection weights are ignored in calculations [69]. It can be defined as follows:

(11) $$\begin{array}{c} k_{i}=\underset{j\in N}{\sum }A_{ij}, \end{array}$$

where *A*_*ij*_ represents the entry *ij* of the adjacency matrix *A*.

Indegree and outdegree of the three judgments (*V*, *T*, and *D*) have been separately compared between valence (+, −), for each band of interest, by performing multiple Student's *t*-test on ROIs, FDR corrected.

### 2.8. Autonomic Data Recording and Signal Processing

The autonomic activity, both the Galvanic Skin Response (GSR) and the Heart Rate (HR), has been recorded by means of the PSYCHOLAB VD13S system (SATEM, Italy) with a sampling rate of 100 Hz. Skin conductance was recorded by the constant voltage method (0.5 V). Ag-AgCl electrodes (8 mm diameter of active area) were attached to the palmar side of the middle phalanges of the second and third fingers of the participant's non dominant hand by means of a velcro fastener. The company also provided disposable Ag-AgCl electrodes to acquire the HR signal. Before applying the sensors to the subjects' skin, their surface has been cleaned following procedures and suggestions published in the international literature [70–72], and GSR and HR signals have been continuously acquired for the entire duration of the stimulation and then filtered and segmented with in-house MATLAB software in order to analyse the autonomic activity related to the observation of the politician faces.

The GSR signal has been downsampled to 20 Hz and subsequently low pass filtered at 4.5 Hz to filter out noise and suppress artefacts caused by Ebbecke waves [70, 73]. In order to split the phasic component of the electrodermal activity (skin conductance response (SCR)) from the tonic one (skin conductance level (SCL)) we acted as follows on the filtered GSR signal:

1. minima points detection within a 100 samples sliding window (5 seconds);
2. linear interpolation of minima points;
3. smoothing by means of a moving average (100 samples sliding window). This operation generates the SCL signal;
4. subtraction of the SCL signal from the filtered GSR. This operation generates the SCL signal.

In such a way, we split the GSR signal into a phasic (SCR) and a tonic (SCL) component and then segmented the traces by taking into account 3 s long segments from the beginning of the face exposition. Afterwards, we generated the same type of datasets as defined for the EEG analysis. As far as concerns the SCL, we calculated the time average for each segment and experimental condition, whereas for the SCR we took into account the average peak number within the data segment. The results of these parameters will be showed in the following section.

The HR signal has been low pass filtered at 1 Hz in order to analyse the frequency components due to variations of the sympathetic and parasympathetic nervous system regardless the ones associated with thermoregulatory cycles [74, 75]. Hence, this waveform has been segmented by taking into account 3 s length segments from the beginning of the face exposition. Afterwards, we generated the corresponding datasets defined for the EEG analysis. From each segment, we calculated the average beats per minute and the power spectrum density (PSD) according to the Welch method [50]. In this way, we obtained a signal in the frequency domain for the all experimental conditions and subjects. Spectral components were identified and then assigned, on the basis of their frequency, to one of two bands: low frequency (LF), [0.04, 0.15] Hz; high frequency (HF), [0.15, 0.6] Hz [37]. The very low frequency (VLF) band, located in the lowest part of the spectrum, has been excluded from the present analysis since it is physiologically connected with long-term regulation mechanisms [74, 75], not of interest for our purpose. Several studies indicate that the LF band corresponds to baroreflex control of the heart rate and reflects mixed sympathetic and parasympathetic modulation of heart rate variability (HRV); instead, HF band corresponds to vagally mediated modulation of HRV associated with respiration [37, 74–76]. For this reason, some researchers [37] propose the ratio LF/HF as index of the balance between the sympathetic and vagal activity.

All autonomic variables of interest (SCL, SCR, HR, and LF/HF) and experimental datasets (*V* ^+^, *V* ^−^, *D* ^+^, *D* ^−^, *T* ^+^, and *T* ^−^) have been standardized by means of the *z*-score transformation by using the dataset referred to the neutral face (*N*) as reference baseline. Repeated-measures analyses of variance (ANOVA) were performed to assess differences in SCL, SCR, HR, and LF/HF. Mauchly's test evaluated the sphericity assumption and, where appropriate correction of the degrees of freedom was made according to the Greenhouse-Geisser procedure. Bonferroni correction was applied to all post hoc tests (pairwise comparisons). The statistical analysis has been performed by the SPSS (v.16) software.

According to the presented methodology, the intersubject variability has been taken into account by computing the *z*-score standardization of the experimental conditions datasets (observation of faces during judgments of vote, dominance, and trustworthiness) and the mean and standard deviation related to the neutral condition (observation of neutral face). Moreover, also the chosen statistical method (paired Student's *t*-test) avoids intersubject variability. Instead, in order to address the interstimuli variability, according to the illustrated experimental paradigm, the single politicians faces have been observed and judged differently by each participant. In this way, each participant has his/her own personal datasets (*V* ^+^/*V* ^−^, *D* ^+^/*D* ^−^, and *T* ^+^/*T* ^−^). In fact, the aim of the present analysis is to investigate the cerebral reaction due to personal judgments rather than objective features and expressions of singles candidates. Hence, data are compared for a population analysis by means of the paired *t*-test.

## 3. Results

### 3.1. Behavioural Results

**Figure 2:**
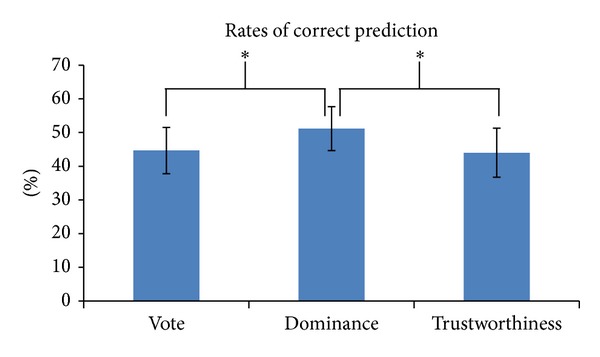
Average rates of correct prediction for the three judgments of vote, dominance, and trustworthiness. Error bars indicate standard deviations. Significant pairwise comparisons are highlighted with asterisks.

In order to asses significant differences among rates of correctly predicted races in the three experimental conditions, we employed a multivariate repeated measures ANOVA design with judgment (vote, dominance, and trustworthiness) as a factor and the percentages of correctly predicted races as a dependent variable. Average values of correct percentages are reported in Figure 2.

The statistical analysis of correct percentages showed a significant difference for the factor judgment across the different levels [vote = (44.65 ± 7.04)%, dominance = (51.15 ± 6.68)%, and trustworthiness = (43.99 ± 7.48)%; *F*(38,2) = 9.34, *P* < 0.01]. Hence, the percentage of correct prediction depends on the judgment. Specifically, the pairwise comparisons revealed significant differences for the contrast vote versus dominance (*P* < 0.01) and dominance versus trustworthiness (*P* < 0.01), whereas no difference has been found between vote and trustworthiness (*P* > 0.05).

Subjects' judgment about vote, dominance, and trustworthiness underwent a correlation analysis. For each pair of judgments (vote/dominance, vote/trustworthiness, and dominance/trustworthiness) we computed the Pearson's correlation among the number of subjects who expressed their preference for the real election winner.

**Figure 3:**
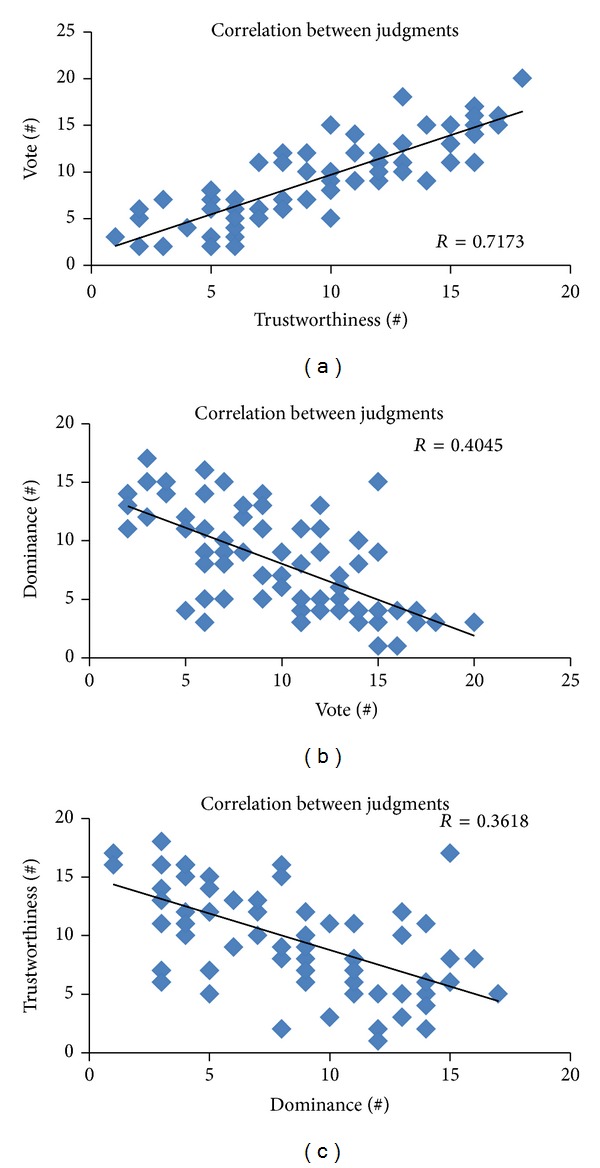
Scatterplots of number of subjects expressing their preference for the real election winner related to judgments of trustworthiness (*x*-axis) and vote (*y*-axis) on (a), vote (*x*-axis) and dominance (*y*-axis) in (b), and dominance (*x*-axis) and trustworthiness (*y*-axis) on (c). Each dot represents a single election race with the corresponding number of subjects who made their choice for the real winner of the election. In each scatterplot, there is the related Pearson's correlation coefficient, all significant at *P* < 0.01.

The results show that judgments concerning vote and trustworthiness are positively correlated (*R* = 0.85, *P* < 0.01) while the ones regarding vote and dominance (*R* = −0.64, *P* < 0.01) and dominance and trustworthiness (*R* = −0.60, *P* < 0.01) are negatively correlated.  Figure 3 shows the correlation between vote and trustworthiness conditions.

### 3.2. Cortical Patterns of Power Spectral Density

The EEG signals gathered during the observation of the politicians were subjected to the estimation of the cortical power spectral density by using the techniques described in the Methods section. In each subject, the cortical PSD was evaluated in the frequency bands adopted in this study and contrasted between experimental conditions. These cortical distributions of PSD obtained during the observation of politicians were then organized in six datasets (*V* ^+^/*V* ^−^, *D* ^+^/*D* ^−^, and *T* ^+^/*T* ^−^). The Student's *t*-test has been performed between these cortical PSD distributions of homologous datasets (i.e., *V* ^+^ versus *V* ^−^). The resulting statistical spectral maps highlight cortical areas in which the estimated PSD statistically differs between two conditions. In the following statistical spectral maps, we show only the results in the theta and alpha frequency bands because they resulted the ones with most activations.

**Figure 4:**
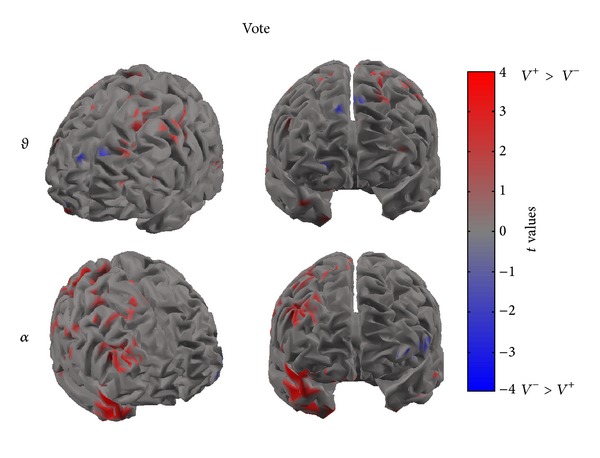
The picture presents four cortical *t*-test maps of PSD values for the vote condition in the theta (upper row) and alpha (lower row) bands. As to the theta band, the cortex model is seen from a front-left side (left) and from a frontal perspective (right). As to the alpha band, the cortex model is seen from a front-right side (left) and from a frontal perspective (right). Colour bar indicates in red cortical areas in which increased statistically significant activity occurs in the *V* ^+^ dataset when compared to the *V* ^−^ dataset. Blue colour is used when the activity is statistically higher in the *V* ^−^ than in the *V* ^+^ condition (*t* values at *P* < 0.05, FDR corrected). Grey colour is used to map cortical areas where there are no significant differences between the cortical activity in the two experimental conditions.

**Figure 5:**
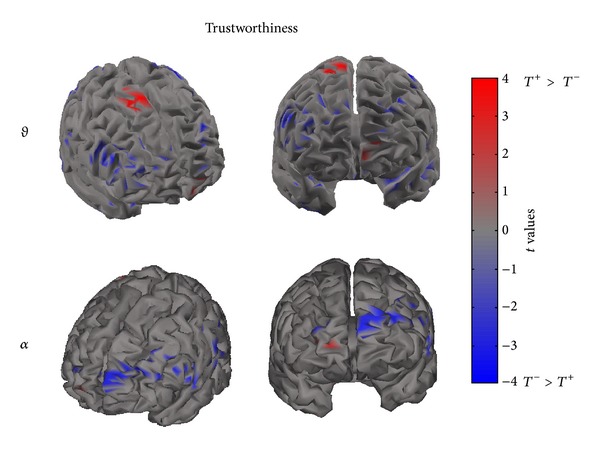
The picture presents four cortical *t*-test maps of PSD values for the trustworthiness condition in the theta (upper row) and alpha (lower row) band. As to the theta band, the cortex model is seen from a front-right side (left) and from a frontal perspective (right). As to the alpha band, the cortex model is seen from a front-left side (left) and from a frontal perspective (right). Colour bar indicates in red cortical areas in which increased statistically significant activity occurs in the *T* ^+^ dataset when compared to the *T* ^−^ dataset. Blue colour is used when the activity is statistically higher in the *T* ^−^ than in the *T* ^+^ condition (*t* values at *P* < 0.05, FDR corrected). Grey colour is used to map cortical areas where there are no significant differences between the cortical activity in the two experimental conditions.

**Figure 6:**
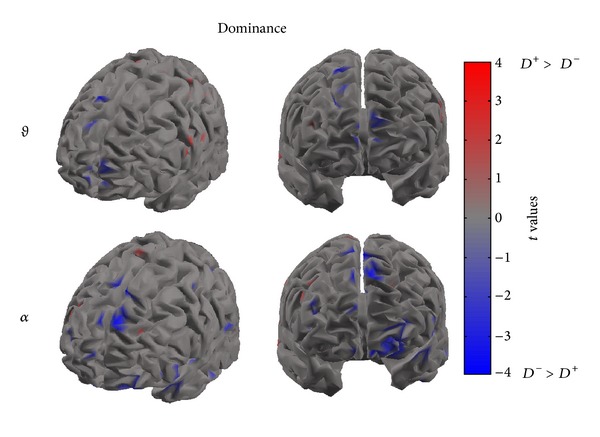
The picture presents four cortical *t*-test maps of PSD values for the dominance condition in the theta (upper row) and alpha (lower row) bands. The cortex model is seen from a front-left side (left) and from a frontal perspective (right) for both theta and alpha bands. Colour bar indicates in red cortical areas in which increased statistically significant activity occurs in the *D* ^+^ dataset when compared to the *D* ^−^ dataset. Blue colour is used when the activity is statistically higher in the *D* ^−^ than in the *D* ^+^ condition (*t* values at *P* < 0.05, FDR corrected). Grey colour is used to map cortical areas where there are no significant differences between the cortical activity in the two experimental conditions.

Figures 4, 5, and 6 present cortical maps in which the brain is viewed from a frontal perspective. The maps are relative to the contrast regarding the conditions vote, dominance, and trustworthiness (i.e., *V* ^+^ versus *V* ^−^) in the frequency bands of interest. The colour scale on the cortex codes the statistical significance: where there are cortical areas in which the power spectrum does not differ between the two conditions, the grey colour is used. The red colour presents statistically significant power spectral activity greater in the condition *V* ^+^(*D* ^+^, *T* ^+^) with respect to *V* ^−^(*D* ^−^, *T* ^−^), while the blue colour codes the opposite situation.

Figure 4 presents the four statistical cortical maps related to the comparisons of PSD values in the theta and alpha bands for the vote condition. These maps highlight a frontal asymmetry which discriminates *V* ^+^ and *V* ^−^ conditions in both theta and alpha bands. Specifically, it is possible to observe an increase of PSD across frontal and central areas in the theta band for the *V* ^+^ condition. Findings in the alpha band return a significant desynchronization for the *V* ^−^ condition in the frontal, central, and parietal cortical regions of the right hemisphere. In addition, a smaller frontal region of the left hemisphere accounts for a desynchronization for the *V* ^+^ condition.

Figure 5 presents the contrast between the *T* ^+^ and *T* ^−^ conditions in the theta and alpha bands. As similarly observed for the vote condition, the comparison between *T* ^+^ and *T* ^−^ also revealed an asymmetrical pattern of activation. In this case, most of the increase of PSD in the theta band is due to the *T* ^−^ condition, involving left frontal cortical areas. However, a significant spot of activation for the *T* ^+^ condition is also visible around right frontal regions. The significant desynchronization of the frontal activity in the alpha band is associated with the *T* ^+^ condition. From Figure 5, it is also possible to observe a sparse activation in left central and temporal areas.

Figure 6 presents the contrast between the *D* ^+^ and *D* ^−^ conditions in the theta and alpha bands. In this case, the significant activations in the theta band show an involvement of the frontal midline regions for the *D* ^−^ condition, whereas a significant desynchronization of the alpha rhythm is visible across left frontal areas for the opposite condition *D* ^+^.

### 3.3. Analysis of Functional Connectivity Patterns and Degree

The two-way ANOVA performed on the density index indicated significant difference for factor BAND for all the three experimental conditions [vote, *F*(3,57) = 4.55, *P* < 0.01; dominance, *F*(3,57) = 4.52, *P* < 0.01; and trustworthiness, *F*(3,57) = 4.23, *P* < 0.01]. This result led us to consider only theta and alpha bands for the following analysis. In fact, both beta and gamma present smaller density values than the lower frequency ranges, and they are close to zero.

The connectivity patterns have been estimated by partial directed coherence (PDC) to the spatially averaged waveforms related to different ROIs considered in the study and statistically compared as described in the Methods section. Student's *t*-tests have been performed between PDC distributions of homologous datasets (i.e., *V* ^+^ versus *V* ^−^). The resulting statistical connectivity maps highlight cortical areas, along with the related strength of the connection, in which the estimated PDC statistically differs between two conditions. Similarly, the analysis of indegree and outdegree has been computed by Student's *t*-test for each ROI and experimental condition.

**Figure 7:**
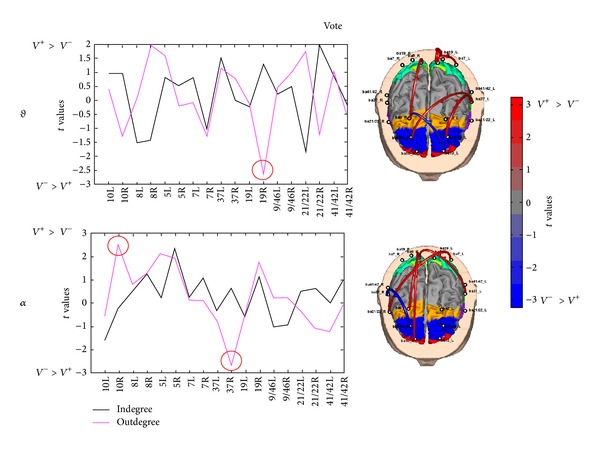
In- and outdegree values (left side) and PDC links (right side) in the theta (upper row) and alpha (lower row) bands for the vote condition. The cortex model is seen from above for both theta and alpha bands. Colour bar indicates PDC connections in which increased statistically significant activity occurs in the *V* ^+^ dataset when compared to the *V* ^−^ dataset in red. Blue colour is used when the increase for the *V* ^−^ activity is statistically higher than the one in the *V* ^+^ condition (*t* values at *P* < 0.05, FDR corrected). The arrows depict the statistically significant connections estimated between the activities recorded in the vote condition. The color and size of the arrows code for the strength of the interaction, as reported by the color bar on the right. A color map highlights the ROIs, whereas grey colour is used to map cortical areas that have not been used in the analysis. Red circles in the left part of the figure highlight significant difference of in- and outdegrees for the theta and alpha bands.

**Figure 8:**
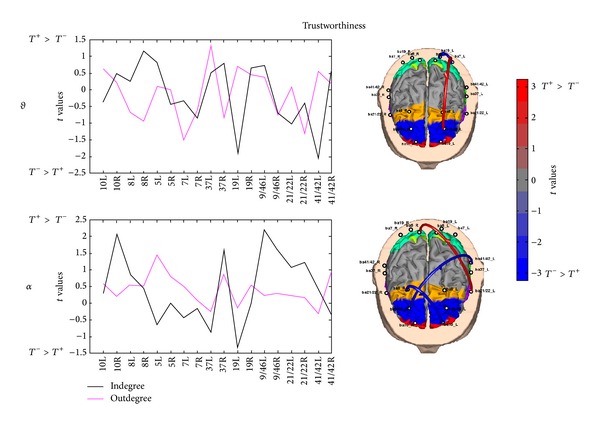
In- and outdegree values (left side) and PDC links (right side) in the theta (upper row) alpha (lower row) bands for the vote condition. The cortex model is seen from above for both theta and alpha bands. Colour bar indicates PDC connections in which increased statistically significant activity occurs in the *T* ^+^ dataset when compared to the *T* ^−^ dataset in red. Blue colour is used when the increase for the *T* ^−^ activity is statistically higher than the one in the *T* ^+^ condition (*t* values at *P* < 0.05, FDR corrected). The arrows depict the statistically significant connections estimated between the activities recorded in the trustworthiness condition. The color and size of the arrows code for the strength of the interaction, as reported by the color bar on the right. A color map highlights the ROIs, whereas grey colour is used to map cortical areas that have not been used in the analysis.

**Figure 9:**
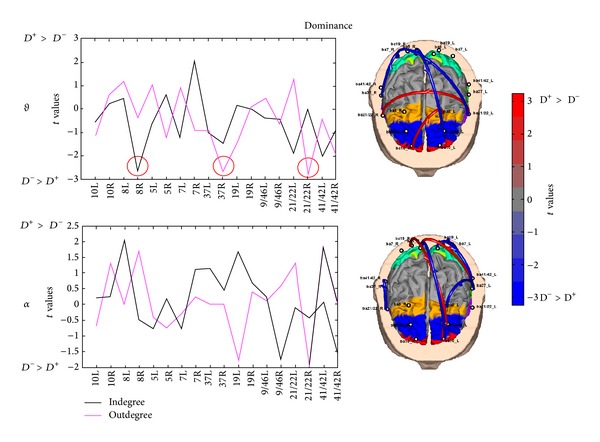
In- and outdegree values (left side) and PDC links (right side) in the theta (upper row) alpha (lower row) bands for the vote condition. The cortex model is seen from above for both theta and alpha bands. Colour bar indicates PDC connections in which increased statistically significant activity occurs in the *D* ^+^ dataset when compared to the *D* ^−^ dataset in red. Blue colour is used when the increase for the *D* ^−^ activity is statistically higher than the one in the *D* ^+^ condition (*t* values at *P* < 0.05, FDR corrected). The arrows depict the statistically significant connections estimated between the activities recorded in the trustworthiness condition. The color and size of the arrows code for the strength of the interaction, as reported by the color bar on the right. A color map highlights the ROIs, whereas grey colour is used to map cortical areas that have not been used in the analysis. Red circles in the left part of the figure highlight significant difference of in- and outdegrees for the theta band.

Figures 7, 8, and 9 present cortical maps, as well as values of indegree and outdegree, in which the brain is viewed from above. The results are related to the contrast regarding the conditions vote, dominance, and trustworthiness (i.e., *V* ^+^ versus *V* ^−^) in the activated frequency bands of interest. The colour scale on the cortex codes the ROIs, and the grey colour is used otherwise. The red colour presents statistically significant connections in the condition *V* ^+^(*D* ^+^, *T* ^+^) with respect to *V* ^−^(*D* ^−^, *T* ^−^), while the blue colour codes the opposite situation.

Figure 7 presents the two statistical connectivity maps related to the comparisons of PDC values in the theta and alpha bands for the vote condition, on the right side of the picture. On the left side, the related comparison for values of in- and outdegrees are reported. These results highlight a strong involvement of frontal and parietal right areas in both theta and alpha bands. Particularly, in the theta band the largest amount of significant inter- and intrahemispheric connections emerges in the left hemisphere related to the *V* ^+^ condition. An interhemispheric flow between the BA10_L and BA8_R is the strongest for the *V* ^−^ condition. From the statistical comparison of in- and outdegrees we may observe that the only significant result highlights the BA19_R as the ROI with highest value of outdegree for the condition *V* ^−^. The statistical pattern in the alpha band presents an involvement of inter- and intrahemispheric connection, thickened in the right hemisphere, in the *V* ^+^ condition. However, a significant connection between BA37_R and BA9/46_R for the *V* ^−^ condition appears. These results are supported by in- and outdegrees values which return the BA10_R as a source of information in the *V* ^+^ condition, whereas the BA37_R source in the *V* ^−^ condition.

Figure 8 presents the two statistical connectivity maps related to the comparisons of PDC values in the theta and alpha bands for the trustworthiness condition, on the right side of the picture. On the left side, the related comparisons for values of in- and outdegrees are reported. These results highlight an involvement of frontal and parietal left areas in both theta and alpha bands although only a few significant connections emerge. Particularly, in the theta band there is a parietal-frontal connection in the *T* ^+^ condition, whereas two parietal BAs are connected in the *T* ^−^ condition. From the statistical comparison of in- and outdegrees, there are no ROIs resulting different between the two experimental conditions in the theta band. The statistical pattern in the alpha band shows the existence of two intrahemispheric connections related to the *T* ^−^ between frontal and parietotemporal regions. There is only one significant link between the BA21/22_L and BA5_R regarding the *T* ^+^ condition. From the statistical comparison of in- and outdegrees, there are no ROIs resulting different between the two experimental conditions in the alpha band.

Figure 9 presents the two statistical connectivity maps related to the comparisons of PDC values in the theta and alpha bands for the dominance condition, on the right side of the picture. On the left side, the related comparison for values of in- and outdegrees are reported. These results highlight a strong involvement of frontal and parietal right areas in both theta and alpha bands. Particularly, in the theta band intrahemispheric parietofrontal connections are related to the *D* ^−^ condition whereas intrahemispheric frontal connections regard the *D* ^+^ condition. From the statistical comparison of in- and outdegrees, we may observe that the BA37_R and BA21/22_R are source of information for the *D* ^+^ condition, whereas BA8_R for the *D* ^−^ condition is a sink. The statistical pattern in the alpha band presents an involvement of inter- and intrahemispheric connection, thickened in the left hemisphere, in both the *D* ^+^ and *D* ^−^ conditions. In particular, the information flow is directed from right parietal to left and temporal frontal areas in *D* ^+^ condition and vice versa for *D* ^−^ condition. From the statistical comparison of in- and outdegrees, there are no ROIs resulting different between the two experimental conditions in the alpha band.

### 3.4. Analysis of the SCL and SCR

To assess the effect of questions on answers, we performed a two-way repeated-measures ANOVA on SCL and on SCR, with judgment (*D*, *T*, and *V*) and valence (+, −) as factors. As far as the SCL is concerned, we did not find significant difference for any factor [judgment: *F*(2,36) = 1.77, *P* = 0.18; valence: *F*(1,18) = 0.04, *P* = 0.83; judgment × valence: *F*(2,36) = 0.63, *P* = 0.54]. However, we also performed Student's *t*-tests to compare pairs of judgments. These statistical analyses returned that the SCL values for the dominance condition are higher than those in trustworthiness [*t*(19) = 2.1, *P* < 0.05] and no difference between the other conditions.

As to the analysis of the SCR, the two-way repeated-measures ANOVA did not highlighted any significant difference for any factor [judgment: *F*(2,36) = 0.21, *P* = 0.82; valence: *F*(1,18) = 1.98,  *P* = 0.18; judgment × valence: *F*(2,36) = 0.18, *P* = 0.80]. However, there is a trend as to the *z*-score of SCR values since for all the six conditions they are always positive with SCR values related to the condition “+” larger than those related to the condition “−”.

### 3.5. Analysis of the HRV

To assess the effect of judgments on the choices of subjects, we performed a two-way repeated-measures ANOVA on HR and LF/HF with judgment (*D*, *T*, *V*) and valence (+, −) as factors.

The two-way ANOVA on the HR parameter did not return any significant result for this comparison [judgments: *F*(2,36) = 1.99, *P* = 0.15; valence: *F*(1,18) = 0.01, *P* = 0.95; judgments × valence: *F*(2,36) = 0.184, *P* = 0.83]. Picture highlights that HR values related to the condition dominance are both negative with respect to the baseline (neutral face), whereas the factor vote presents positive values. As to the factor trustworthiness, the conditions “+” and “−” have a positive (0.01) and negative (−0.03) values, respectively. However, we also performed Student's *t*-tests to compare pairs of judgments. These statistical analyses returned that the HR values for the vote condition are higher than those in dominance [*t*(19) = 2.11, *P* < 0.05] and no difference between the other conditions.

The two-way ANOVA on the LF/HF parameter did not return any significant result for this comparison [judgment: *F*(2,36) = 1.47, *P* = 0.24; valence: *F*(1,18) = 0.66, *P* = 0.43; judgment × valence: *F*(2,36) = 0.12, *P* = 0.99]. In each condition, the average value of the sympathovagal balance is negative. This means that the LF/HF values are always larger during the observation of the neutral face compared to the exposition of politicians. Moreover, the LF/HF values related to the condition dominance are the smallest ones. However, we also performed Student's *t*-tests to compare pairs of judgments. These statistical analyses returned that the HR values for the trustworthiness condition are lower than those in dominance [*t*(19) = 2.1, *P* < 0.05] and no difference between the other conditions.

## 4. Discussion

### 4.1. Explicit Judgment

The analysis conducted on the participant's choice returned low percentages of correct prediction for all the three possible judgments. Two of them are below the 50% (vote, 44.6%; trustworthiness, 43.9%) while the only judgment about dominance shows a percentage of 51.15. In fact, for our subjects, the judgment on the dominance trait resulted the most predictive, with respect to trustworthiness and preference of vote, of the election outcome. This result could appear contrasting the findings previously published in the literature [1, 2], since they reported a goodness of prediction around 70% in guessing the elections outcome. This difference could be due to the exiguous number of participants enrolled in our study (20) if compared to the number of subjects utilized in their previous researches (more than 100). In fact, a more recent study performed with fMRI [5] enrolling 24 participants reported lower percentage in judging winners of real elections as more competent (55%). This evidence could suggest a positive correlation between the traits of dominance and competence employed in the two studies. Although Oosterhof and Todorov [40] established mean ratings for computer modelled faces on 14 trait dimensions, showing a certain correlation among them, the trait of competence is not in the trait set used for the study. Hence, we do not discuss about the degree of correlation between the traits of competence and dominance. However, in the same work, traits of dominance and trustworthiness appear to be orthogonal. Instead, dominance appears to be correlated with aggressiveness. Hence, it was not surprising for us to get a similar result for real faces. This kind of dependence could explain the positive result in predicting winners as more dominant and the negative result in prediction about the trustworthiness judgment. However, it is important to remember that our participants were asked to give their judgments according to a fast exposition of unknown face of politicians. Therefore, they do not observe more than a few physical traits and know nothing related to their public behavior.

The correlation analysis concerning answers about the three judgments revealed that our experimental subjects tend to vote politicians appearing more trustworthy. However, we observed that this judgment is not able to predict elections outcomes.

### 4.2. Cortical Patterns of Power Spectral Density and Functional Connectivity

The high-resolution EEG analysis returned statistically significant activations in all experimental conditions of vote, dominance, and trustworthiness judgments. Overall, evidence highlights an asymmetrical cerebral activation, mostly related to the frontal areas, during the observation of politicians that will be judged trustworthy and dominant and for those who will get the vote in simulated elections. Thus, such neuroelectrical features seem to be able to predict judgments of trustworthiness and dominance as well as the decision of vote.

Particularly, the vote condition elicited the most significant variations of the PSD in the alpha band. The related cortical maps show a significant desynchronization of the alpha rhythm among frontal and parietal areas of the right hemisphere during the observation of politicians that have not been voted in our simulated elections, along with a smaller increase of PSD in left frontal areas for the opposite condition. Analogously, the pattern of activations in the alpha band for the trustworthiness condition show a desynchronization of the left frontal regions associated with the observation of candidates that will be judged more trustworthy. Instead, looking at the cortical maps in the theta band in both experimental conditions, it is possible to appreciate almost a reverse behavior of the frontal regions. Specifically, left frontal areas are more involved during the observation of candidates that will be voted whereas right frontal areas are more activated during the observation of politicians that will be judged less trustworthy. Hence, the asymmetrical pattern of activation in these particular tasks seems to concern both theta and alpha bands. In the dominance condition, it is possible to observe that the desynchronization of the left frontal regions is related to the observation of candidates that will be judged more dominant. Adversely, the activations in the theta band mostly regard the frontal midline which results activated during the observation of politicians resulting less dominant.

Previous studies have shown that the frontal cortex (FC) is anatomically and functionally connected to structures linked to the emotional processing activity in humans [77]. Thus, indirect variables of emotional processing could be also gathered by tracking variations of these cerebral cortical frontal areas. In fact, although the frontal cortex is structurally and functionally heterogeneous, its role in the generation of the emotions is well recognized [78]. EEG spectral power analyses indicate that the anterior cerebral hemispheres are differentially lateralized for approach and withdrawal of motivational tendencies and emotions. Specifically, findings suggest that the left frontal and orbitofrontal cortex is an important brain area in a widespread circuit that mediates appetitive approach, while the right homologues regions appear to form a major component of a neural circuit that instantiates defensive withdrawal [79, 80].

Sutton and Davidson [81] found that greater left-sided activation predicted dispositional tendencies toward approach, whereas greater right-sided asymmetry predicted dispositional tendencies toward avoidance. In contrast, their frontal asymmetry measurement did not predict dispositional tendencies toward positive or negative emotions, suggesting an association of frontal asymmetry with approach avoidance rather than with valence. Other sources of data converge on a similar model of frontal asymmetry. Of particular importance are studies that link anger, an unpleasant but approach-related emotion, to greater left-hemispheric activation [82, 83]. Also, tendencies toward worry, thought to be approach-motivated in the sense of being linked to problem solving, have been linked to relatively greater left frontal EEG activity [84]. Thus, the emerging consensus appears to be that frontal EEG asymmetry primarily reflects levels of approach motivation (left hemisphere) versus avoidance motivation (right hemisphere), as also testified by previous studies [85–88].

Hence, the present results could be interpreted by taking into account the approach-withdrawal theory [78–80] explaining that there exists an EEG asymmetry, mostly involving frontal regions, discriminating appealing, pleasantness situations from unattractive and unpleasant ones: a desynchronization of the alpha rhythm in left frontal areas is experienced when subjects are involved in positively judged contexts, whereas a desynchronization of same areas in the right hemisphere is often associated with rejecting experiences. In addition, our experiment also highlights a frontal-hemispheric asymmetry involving the theta band. Therefore, the faces of politicians that will be voted and judged trustworthy arise feelings of approach, whereas those candidates that will be judged less trustworthy and not adequate for winning the ballot generate emotions of detach and refusal. This interpretation emerges from considering the activations in both theta and alpha bands and is in agreement with the behavioral results providing a high and significant correlation between the judgment of trustworthiness and the choice of vote. When subjects expressed judgment on dominance trait, politicians judged more dominant elicited an alpha desynchronization across left frontal and orbitofrontal areas. As anger, also the trait of dominance could be considered as an approach-related emotion, with a negative valence and high arousal in a tentative to reconcile the concept of discrete emotions in terms of combinations of multiple dimensions [89, 90]. According to this perspective, the alpha activity in the right hemisphere is in agreement with the approach-withdrawal theory. On the contrary, low dominance could lead to withdrawal-related emotions with positive valence and low arousal. Observation of politicians judged less dominant caused a significant activation of the medial frontal cortex (mFC) in the theta band. Such signals could be connected to the activity of the anterior cingulate cortex (ACC). In fact, the circuit ACC-mFC is involved in processing emotions. In particular, Phan et al. [91] reported that 60% of the studies they reviewed found activation in the medial frontal cortex (mFC), whereas Murphy et al. [92] reported the strongest localization pattern in the supracallosal ACC, both related to sadness which is a low arousal emotion. Moreover, the link between frontal midline and the activity in the anterior cingulate cortex is also shown through listening to pleasant music since ACC is activated in musical tasks [93, 94]. This evidence shows that ACC could be involved in processing low arousal, pleasant, and withdrawal-related emotions. Our result regarding the dominance trait and the activation of ACC is also in agreement with the study by Spezio et al. [5] showing that this specific brain area is a predictor of the simulated election loss. In fact, the observation of politicians judged less dominant elicited an increase of activity in the frontal midline which is negatively correlated with the lab vote share. In addition, the activation related to the dominance condition could reflect neural processing to disgusted facial expressions and angry faces which exhibit involvement of the right putamen and the left insula cortex, enhancing activity in the posterior part of the right gyrus cinguli and the medial temporal gyrus of the left hemisphere. Fearful expressions also activate the right fusiform gyrus and the left dorsolateral frontal cortex [95, 96].

The role of prefrontal cortex is also in agreement with the experiment performed by Kato and colleagues [6] that illustrate how stronger fMRI activation in the dorsolateral prefrontal cortex lowered ratings of candidates originally supported more than did those with smaller fMRI signal changes in the same region. On the contrary, the subjects showing stronger activation in the medial prefrontal cortex tended to increase their ratings compared to subjects with less activation.

The involvement of left ventral frontal cortex can be also associated with neural response to facial emotions, regardless of the conscious mediation [97]. In addition, the activity of the temporal lobe has already been reported belonging to judgments of trustworthiness trait [98].

Although it has not been investigated in the present study, it could be debated that the discussed patterns of activation may vary according to the gender. In fact, previous studies dealing with emotions processing report that females respond strongly than males to emotional and negative faces, judged unpleasant and high arousing stimuli. As reported by Lithari et al. [99], these differences involve mechanisms localized across central and left brain regions. Supporting this evidence, Prause et al. [100] found that women elicited a stronger frontal alpha asymmetry during the observation of sexually motivating films, with a greater alpha power in the left hemisphere. Similar gender differences have been also specifically inspected during judgment of facial attractiveness [101]. In fact, Zhang and Deng report a delayed P1 and P3b latencies in response to attractive faces with slower response times in men compared to women. A further study also shows that women tend to prioritize the processing of socially relevant and negative emotional information [102]. Overall, such results summarize that emotion processing mostly involve central and left frontal regions both for men and women, although the latter does it with a stronger intensity and smaller latency. Instead, an important functional gender differentiation could be investigated across the right hemisphere [103]. In fact, right-lateralized anticipatory activity selectively influenced the encoding of unpleasant pictures in women but not in men. These findings indicate that anticipatory processes influence the way in which women encode negative events into memory. The selective use of such activity may indicate that anticipatory activity is one mechanism by which individuals regulate their emotions, also in face processing although this affirmation needs to be confirmed by further experiments.

### 4.3. Autonomic Variables

As far as the results of the electrodermal activity are concerned, we reported that the tonic component of the galvanic skin response is significantly lower while participants observed politicians during the trustworthiness judgment when compared with the judgment of dominance and vote. Specifically, SCL in the trustworthiness condition is lower than the average value elicited during the observation of a neutral face, whereas SCL in dominance and vote conditions is higher than that recorded in the same baseline condition. According to Critchley [35, 104], the tonic level of the galvanic skin response decreases during attentional tasks. The SCL elicited in our experimental task varies according to the judgment to be expressed. Since the judgment about trustworthiness returned lower SCL values, it seems that this task is more demanding if compared to the question of dominance and vote. Instead, as to the phasic component of the galvanic skin response, we did not find any significant difference from the statistical point of view. However, by observing the average peak amplitude of the SCR, values related to the choice of subjects appear higher than values associated with politicians that have not been chosen, though not statistically significant. Hence, the observation of politicians that will be later judged, irrespective of the proposed trait, could induce a stronger emotional state reflected in a variation of the autonomic nervous system.

As to the analysis of the HRV, statistical tests performed on average values of heart rate returned an increase of the heart rate during the observation of politicians to be voted, whereas the related values in the dominance and trustworthiness judgments are negative when compared to the observation of the neutral face. Also, the sympathovagal balance returned negative values for all the experimental conditions when compared to the baseline with an increase for the dominance condition with respect to the trustworthiness. Hence, the activity of the parasympathetic nervous system seems to be prevailing during the observation of politicians to be judged trustworthy. There are also several studies reporting the increment of the vagal tone during states of sustained attention [100] in agreement with the result of the skin conductance level reporting an increase of attention for the judgment of trustworthiness with respect to the trait of dominance. In addition, the modulation of the HRV is also correlated with attentional engagement to fearful faces [101] and emotional face processing [102] which could be in line with the results related to the observation of politicians during the judgment of dominance.

Overall, autonomic results suggest that trait judgments of dominance and trustworthiness are related to visceral responses in terms of skin conductance level and vagal tone.

## 5. Conclusions

The use of the high-resolution EEG techniques [105–112] in an evaluation of the efficacy of trustworthy and dominant faces of political candidates has generated interesting results. Such findings suggested the following answers to the questions elicited in introduction.

1. For the analysed population, we found out that the judgment on the trait of dominance after a rapid observation of politicians' faces is the most predictive, with respect to the one of trustworthiness and preference of vote, of the election outcome. Preferences of trustworthiness and vote are positively correlated.
2. By analysing the PSD cortical maps and the related PDC connectivity patterns, the results highlighted frontal asymmetrical activities characterizing all the experimental conditions of vote, trustworthiness, and dominance. These findings are in agreement with the approach-avoidance theory and can predict the decision of vote, as well as the judgment of trustworthiness and dominance.
3. Despite not being completely statistically significant, the analysis of the autonomic parameters revealed a decrease of skin conductance level and sympathovagal balance related to the observation of politicians to be judged as trustworthy related to an increase of sustained attention. These features could correlate with modulation of attention and emotional engagement to faces.
